# A phase I study of selinexor in combination with high-dose cytarabine and mitoxantrone for remission induction in patients with acute myeloid leukemia

**DOI:** 10.1186/s13045-017-0550-8

**Published:** 2018-01-05

**Authors:** Amy Y. Wang, Howard Weiner, Margaret Green, Hua Chang, Noreen Fulton, Richard A. Larson, Olatoyosi Odenike, Andrew S. Artz, Michael R. Bishop, Lucy A. Godley, Michael J. Thirman, Satyajit Kosuri, Jane E. Churpek, Emily Curran, Kristen Pettit, Wendy Stock, Hongtao Liu

**Affiliations:** 1 Internal Medicine/Pediatric Residency Program, University of Chicago Medicine, Chicago, IL USA; 2 Department of Medicine, Section of Hematology/Oncology, University of Chicago Medicine, 5841 S. Maryland, MC 2115, Chicago, IL 60637-1470 USA; 3 Karyopharm Therapeutics Inc, 85 Wells Avenue, Suite 210, Newton, MA 02459 USA; 4 Department of Medicine, Section of Hematology/Oncology, University of Michigan Medicine, Ann Arbor, MI USA

**Keywords:** AML, Selinexor, Induction chemotherapy, XPO1/CRM1

## Abstract

**Background:**

Novel therapies for patients with acute myeloid leukemia (AML) are imperative, particularly for those with high-risk features. Selinexor, an exportin 1 (XPO1/CRM1) inhibitor, has demonstrated anti-leukemia activity as a single agent, as well as in combination with anthracyclines and/or DNA-damaging agents.

**Methods:**

We report the findings of a phase I dose escalation trial with cohort expansion in 20 patients with newly diagnosed or relapsed/refractory AML that combined selinexor with age-adjusted high-dose cytarabine and mitoxantrone (HiDAC/Mito).

**Results:**

Three (15%) patients received the initial dose of 60 mg of selinexor (~ 35 mg/m^2^), and 17 (85%) received the target level of 80 mg (~ 50 mg/m^2^). No dose-limiting toxicities were observed. Common adverse events included febrile neutropenia (70%), diarrhea (40%), anorexia (30%), electrolyte abnormalities (30%), bacteremia (25%), cardiac toxicities (25%), fatigue (25%), and nausea/vomiting (25%). None were unexpected given the HiDAC/Mito regimen. Serious adverse events occurred in 6 (30%) patients; one was fatal. Ten (50%) patients achieved a complete remission (CR), 3 (15%) achieved CR with incomplete recovery (CRi), 1 (5%) achieved partial remission (PR), and 6 (30%) had progressive disease for an overall response rate (ORR) of 70%. Eight of 14 (57%) responders proceeded to allogeneic stem cell transplantation. Correlative studies of WT1 levels showed persistently detectable levels in patients who either did not respond or relapsed quickly after induction.

**Conclusion:**

The selinexor/HiDAC/Mito regimen is feasible and tolerable at selinexor doses of 80 mg/day (~ 50 mg/m^2^/day) twice weekly. The recommended phase II dose is 80 mg and warrants further study in this combination.

**Trial registration:**

ClinicalTrials.gov, NCT02573363. Registered October 5, 2015

## Background

Acute myeloid leukemia (AML) is the most common acute leukemia in adults and is characterized by a poor prognosis with a 5-year survival rate of approximately 30% [1]. Even patients who achieve remission to initial therapy often relapse, and certain subgroups such as those with therapy-related AML, older patients, and those with relapsed or refractory disease have a particularly poor outcome [2, 3]. Novel agents that are active and able to be widely applied are imperative. Selinexor (KPT-330), an exportin 1 (XPO1) inhibitor, may be such an agent.

XPO1 is the exclusive, nuclear exporter of most major tumor suppressor proteins (TSP) and growth regulatory proteins (GRP), including p53, p21, p73, FOXO1, and NPM1 [4–6]. In leukemia and solid tumors, XPO1 is overexpressed leading to enhanced transport of these proteins to the cytoplasm, thereby neutralizing their anti-neoplastic functions and functionally inactivating TSP/GRP. Elevated levels of XPO1 have been independently associated with a worse prognosis in adults with AML [7, 8]. These findings presented an attractive target for the development of a novel class of XPO1 inhibitors. However, the earliest compounds of XPO1 inhibitors had faced significant toxicities in phase I clinical trials requiring discontinuation, while others were never studied in a clinical setting [9].

Selinexor (KPT-330) is an oral, first-in-class, slowly reversible, and potent agent that was among the next group of selective inhibitor of nuclear export (SINE) compounds to be developed, which also included KPT-335, KPT-185, KPT-276, and KPT-251, many of which have since been studied in clinical trials. The SINE compounds bind to residue Cys528 of XPO1 and blocks the transport of cargo proteins [5]. In both AML primary samples and murine xenograft models, SINE compounds have been shown to reduce XPO1 levels and enhance the nuclear accumulation of p53 [10, 11]. A phase I clinical trial of selinexor in patients with advanced AML demonstrated it to have a manageable safety profile at 35 mg/m^2^ (60 mg) and to be efficacious in a single-agent setting [12].

In vivo and in vitro evidence suggest synergistic activity against leukemic cells by combining selinexor with anthracyclines or DNA-damaging agents [13]. The combination of high-dose cytarabine (HiDAC) with the anthracycline mitoxantrone (Mito) is an effective induction regimen for patients with AML [14–17] and is frequently utilized at the University of Chicago as frontline therapy for patients with high-risk AML, either de novo or relapsed/refractory. The HiDAC/Mito regimen demonstrated an overall response rate (ORR) of 55% in the high-risk AML population at this institution [16] and an ORR of 82% in another study of previously untreated patients with therapy-related myeloid neoplasms [17].

Based on phase I safety and efficacy data of selinexor in patients with AML, the promising results with HiDAC/Mito induction, and the in vivo and in vitro data suggesting synergistic killing of AML cell lines by combining selinexor and anthracyclines, we hypothesized that selinexor would sensitize AML cells to the cytotoxic effects of HiDAC/Mito. We monitored the impact on minimal residual disease (MRD) by tracking the level of Wilms tumor 1 (WT1) expression. WT1 levels in the peripheral blood predict relapse after remission, and their levels after consolidation therapy are closely correlated with survival and early relapse [18, 19]. In this phase I study, we describe the safety, tolerability, correlative molecular studies, and activity of selinexor in combination with HiDAC/Mito for remission induction in patients with previously untreated or relapsed/refractory (R/R) AML.

## Methods

### Study subjects and design

We performed a phase I dose escalation trial with cohort expansion that combined increasing doses of selinexor with age-adjusted HiDAC/Mito (NCT02573363). The trial was approved by the Institutional Review Board at The University of Chicago (IRB15-0412). Patients with newly diagnosed or R/R AML, except acute promyelocytic leukemia, were eligible to enroll if they had adequate performance status (ECOG ≤ 2), cardiac function (ejection fraction > 50%), renal function (creatinine clearance > 30cm^3^/min), and hepatic function (transaminases ≤ 3.0 times upper limit of normal). Exclusion criteria included active central nervous system (CNS) leukemia, pregnancy, recent treatment with any investigational agent, recent major surgery, concurrent malignancy under active treatment, active infection, or recent seizure or stroke. Dose escalation of selinexor was performed according to a 3 + 3 design [20]. Patient enrollment began in October 2015.

### Treatment regimen

**Fig. 1:**
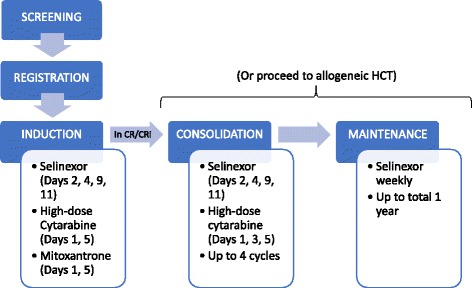
Study schematic. Schematic of phase 1 dose escalation study. Selinexor dosing remained unchanged for all phases, except for 2 patients who received reduced selinexor dosing during consolidation. Twenty patients entered induction, and 14 achieved a response. No patients received a second cycle of induction. No patients withdrew. Six patients entered consolidation, but 1 soon proceeded to allogeneic HCT. Ultimately, 8 underwent allogeneic HCT. One patient quickly relapsed after induction prior to starting the next phase of therapy. HCT = hematopoietic cell transplantation

Figure 1 shows the study schematic. HiDAC (3 g/m^2^, or 2 g/m^2^ if > 70 years, intravenously over 4 h) followed immediately by Mito (30 mg/m^2^, or 20 mg/m^2^ if > 70 years, intravenously over 1 h) were administered on days 1 and 5. This regimen was based on several studies demonstrating efficacy in response and leukemia cell dynamics [14, 15]. Selinexor was given orally on days 2, 4, 9, and 11. Initial selinexor dose was 60 mg (~ 35 mg/m^2^ for an average adult) followed by dose escalation to a target level of 80 mg (~ 50 mg/m^2^). Patients who failed to achieve remission after induction were taken off the study. A second cycle of induction was not given. Patients who entered remission proceeded to allogeneic hematopoietic cell transplantation (HCT), if feasible, or consolidation chemotherapy with HiDAC and selinexor for up to four cycles at the same dose, followed by maintenance therapy with weekly selinexor for up to 1 year. Dose adjustments were allowed during consolidation or maintenance if patients experienced grade > 3 toxicities. The selinexor doses and schedule were determined based on results from prior phase I studies in hematologic malignancies (KCP-330-001 [NCT01607892]), solid tumors (KCP-330-002 [NCT01607905]), and sarcomas (KCP-330-003 [NCT01896505]). These studies demonstrated a maximum tolerated dose (MTD) of 80–120 mg given twice weekly over a 4-week cycle but recommended a lower dose at 60–80 mg to better enable successful chronic use. Prophylactic antimicrobial and anti-emetic agents were administered according to institutional guidelines.

### Toxicity assessment

Patients in the dose escalation cohorts were monitored for dose-limiting toxicities (DLT) for up to 56 days. DLT was defined as any grade ≥ 3 treatment-related non-hematologic toxicity, or persistent bone marrow aplasia in the absence of AML, or any grade 4 neutropenia or thrombocytopenia at day 56 of induction therapy not explained by disease recurrence or infection. Once a dose level was declared tolerable, more patients could be enrolled at that level to provide additional safety, tolerability, and efficacy data. Patients in the dose expansion cohort were closely monitored for adverse events (AE) but not assessed for DLT per the study design. All AEs were noted, including any serious adverse events (SAE), which were AEs that resulted in death, were life-threatening (patient is at immediate risk of death from the event as it occurred), required inpatient hospitalization or prolongation of existing hospitalization for ≥ 24 h, resulted in significant disability/incapacity, or resulted in a congenital anomaly or birth defect.

### Response criteria and monitoring

The primary objective was to determine the recommended phase II dose (RP2D) of selinexor with HiDAC/Mito. Secondary objectives were to determine the complete remission (CR) rate, toxicities, progression-free survival (PFS) and overall survival (OS) rates, and allogeneic HCT success rate, defined as the number of patients who proceeded to HCT following remission. CR was defined as being transfusion independent with an absolute neutrophil count (ANC) > 1.0 × 10^9^/L, platelet count > 100 × 10^9^/L, and bone marrow blasts < 5% [21, 22]. CR with incomplete recovery (CRi) was defined as meeting all CR criteria except for residual neutropenia (< 1.0 × 10^9^/L) or thrombocytopenia (< 100 × 10^9^/L). Partial remission (PR) was defined as a decrease in pre-treatment bone marrow blast percentage by at least 50% and to within the range of 5–25%, while otherwise meeting all hematologic criteria of CR. Lastly, a treatment failure (TF) was defined as resistant disease, relapse, or death [22]. All patients were followed until disease progression, withdrawal, death, or up to 1 year after completion of all protocol treatment.

### Correlative molecular studies

To assess WT1 levels as a marker of MRD, peripheral blood and marrow aspirate samples were collected before induction, at day 12, at the patient’s blood count recovery or prior to day 56, and again at relapse. Bone marrow aspirate and/or peripheral blood mononuclear cell cDNA samples and template controls were assayed by quantitative real-time PCR (qRT-PCR) analysis utilizing Taqman gene expression assays (Life Technologies) for *WT1* (Hs01103751) and *ABL* (Hs01104728) in triplicate using LightCycler 48011 (Roche). All transcript expression levels were determined by reference to standard curves generated from fivefold serial dilutions of K562 cell line cDNA (0.08–250 ng). The absolute transcript copy number was normalized to the endogenous control gene, *ABL1*.

To demonstrate the effect of selinexor with HiDAC/Mito on the bone marrow, routine hematoxylin and eosin (H&E) and immunohistochemistry (IHC) staining for Ki67, p53, SMAD4, Rb, and p21 were performed with formalin-fixed paraffin-embedded sections cut at 5 μm. For IHC staining, slides were baked at 65 °C for 30 min, processed for deparaffinization and rehydration, and then placed in Declare working buffer, steam-cooked for antigen retrieval, cooled, and transferred to 3% hydrogen peroxide to block endogenous hydrogenase activity. Protein block was applied before primary antibodies were incubated with slides. Cell Marque Hi-Def Polymer Amplifier and Secondary Antibody were applied sequentially at room temperature as per manufacturer’s instructions. DAB chromogen was used for color reaction. Slides were counterstained with hematoxylin, dehydrated, mounted, and cover-slipped. IHC staining was performed on a Biogenex I6000 automated stainer. Digital images of the slides were obtained through an Aperio AT Turbo image scanner at × 20.

### Statistics

Survival rates were calculated using the Kaplan-Meier method in the statistical software R (version).

## Results

### Patient characteristics

Twenty patients enrolled and received selinexor with HiDAC/Mito. Table 1 lists the patient characteristics. Three patients received selinexor 60 mg, and 17 patients received 80 mg. Fourteen (70%) patients were female. The median age was 61 with a range of 44–75 years. Twelve (60%) patients had untreated AML, and 8 (40%) patients had R/R disease. Twelve (60%) patients were diagnosed with de novo AML, and 8 (40%) patients had AML after a myelodysplastic syndrome (MDS). There were no patients with therapy-related AML. Four (20%) patients had favorable risk, 8 (40%) had intermediate risk, and 8 (40%) had adverse risk AML by European LeukemiaNet criteria. Five (25%) patients had *FLT3* mutations; 3 were internal tandem duplications (ITD), and 2 were point mutations in the tyrosine kinase domain (TKD). Two (10%) patients had *CEBPA* mutations, and 5 (25%) patients had *NPM1* mutations. The median number of prior regimens for the R/R patients was 2 and included cytarabine with anthracycline (7 + 3), HiDAC, hypomethylating agents, tyrosine kinase inhibitors, FLAG-IDA, ATRA (for EV1 translocation), and other investigational agents.

**Table 1:** Characteristics of the patients

Patient characteristics	Number (%)
Total patients enrolled	20
Selinexor 60 mg	3
Selinexor 80 mg	17
Female	14 (70%)
Median age (years, range)	61 (44–76)
Disease state on enrollment	
Untreated AML	12 (60%)
Relapsed or refractory AML^a^	8 (40%)
Initial AML diagnosis	
De novo AML	12 (60%)
Secondary AML after MDS	8 (40%)
European LeukemiaNet genetic risk group	
Favorable	4 (20%)
Intermediate I/II	8 (40%)
Adverse	8 (40%)
Acquired mutation status	
FLT3	3 (15%) with ITD, 2 (10%) with TKD mutation
CEBPA	2 (10%) (one had bi-allelic mutation)
NPM1	5 (25%) (3 with FLT3 mutation)
Median number of prior regimens (R/R only)^a^	2 (range, 1–3)

*ITD* internal tandem duplication, *TKD* tyrosine kinase domain

^a^Prior therapies include cytarabine with anthracycline (7 + 3), HiDAC, hypomethylating agents (decitabine), tyrosine kinase inhibitors, FLAG-IDA, ATRA (for EV1 translocation), and investigational agents

### Toxicity

Myelosuppression was the most common hematologic toxicity and was universal. However, patients had longer than expected duration of neutropenia and thrombocytopenia. The median time to achieve CR was 37.5 days (range 26–50 days). The median time to achieve an ANC > 0.5 × 10^9^/L was 31 days (range 22–48 days) and platelet count > 20 × 10^9^/L was 25 days (range 19–38 days).

Table 2 lists the non-hematologic adverse events observed during induction, consolidation, and maintenance phases, of which febrile neutropenia was the most common at 70%. No patients required intensive care because of infection. Gastrointestinal toxicities were common: diarrhea (40%), anorexia (30%), and nausea and vomiting (25%). Most were grade 1 or 2 and manageable with supportive therapies. Other frequent adverse events included electrolyte abnormalities (hyponatremia or hypokalemia in 30%), bacteremia (25%), cardiac toxicities (25%), fatigue (25%), pneumonia (20%), and alopecia (20%). Cardiac toxicities included 2 patients with reduced ejection fraction (1 had prior history of anthracycline exposure), 1 with atrial fibrillation, 1 with right bundle branch block, and 1 with prolonged QT interval due to anti-emetics. SAEs occurred in 6 (30%) patients, which included a severe urinary tract infection (during maintenance), cerebellar toxicity, hemorrhagic stroke, cellulitis (during consolidation), endocarditis, and intractable nausea/vomiting.

**Table 2:** Adverse events observed in > 5% of patients

Adverse events	Total, *n* (%)	Grades 1 and 2	Grade 3	Grade 4	Grade 5
Febrile neutropenia	14 (70%)		14		
Diarrhea	8 (40%)	8			
Anorexia	6 (30%)	6			
Electrolyte abnormalities	6 (30%)	6			
Bacteremia	5 (25%)		5		
Cardiac toxicity^b^	5 (25%)	2	3		
Nausea/vomiting	5 (25%)	4	1^a^		
Fatigue	5 (25%)	5			
Pneumonia	4 (20%)		4		
Alopecia	4 (20%)	4			
Line-associated DVT	3 (15%)		3		
Acute kidney injury	3 (15%)	3			
Rash	3 (15%)	3			
Mood disorders	3 (15%)	3			
Clostridium difficile colitis	2 (10%)	2			
Syncope/pre-syncope	2 (10%)	1	1		
Upper respiratory infection	2 (10%)	2			
Mucositis	2 (10%)	2			
Transaminitis	2 (10%)	2			
Psychosis	2 (10%)	2			
Typhlitis	1 (5%)		1		
Hypoxia	1 (5%)		1		
Urinary tract infection	1 (5%)		1^a^		
Cerebellar toxicity	1 (5%)		1^a^		
Hemorrhagic stroke	1 (5%)				1^a^
Cellulitis	1 (5%)		1^a^		
Endocarditis	1 (5%)		1^a^		
Total	93	55	37	0	1
	Adverse events occurring ≤ 5% not listed above: diverticulitis, edema, dysuria, musculoskeletal pain, vaginitis, plantar fasciitis, dry mouth, dysphagia, otitis externa, conjunctivitis, gingivitis, chest pain, hyperbilirubinemia, hypoalbuminemia, INR increased, peripheral neuropathy, and insomnia.				

*DVT* deep vein thrombosis; *INR* international normalized ratio

^a^Serious adverse event

^b^Cardiac toxicities included reduction in ejection fraction (2), atrial fibrillation (1), sinus bradycardia (1), and prolonged QT interval (2)

No DLT was observed during the dose escalation phase. However, 3 patients in the subsequent expansion cohort had noteworthy toxicities: one patient with a hypocellular bone marrow prior to induction had an aplastic marrow lasting beyond day 56; another suffered a hemorrhagic stroke with platelet count of 4000/μL and died during induction; a third patient required total parenteral nutrition (TPN) briefly due to severe nausea, vomiting, and anorexia, despite supportive therapies.

### Response

Of the 20 patients evaluable for response, 10 (50%) achieved CR, 3 (15%) achieved CRi, 1 (5%) achieved PR, and 6 (30%) had TF (Table 3). The patient who achieved PR received selinexor 60 mg monotherapy twice a week for 2 weeks through an approved protocol amendment and achieved CRi, which allowed the patient to proceed to a HCT. The overall response rate (ORR) was 70% (*n* = 14/20). Patients who received selinexor 80 mg (*n* = 17) had an ORR of 76%; the ORR was 33% for patients who received 60 mg (*n* = 3). The ORR was 80% for younger patients (age ≤ 60) and 50% in older patients. Patients with newly diagnosed AML had an ORR of 92% (vs 38% for R/R). All patients with favorable risk achieved CR, whereas the intermediate and adverse risk groups achieved an ORR of 67 and 63%, respectively. Statistical significance was not calculated due to small sample size.

**Table 3:** Responses to the treatment

Response rates	Total	CR (%)	CRi (%)	PR (%)	TF (%)	ORR (%)
Dose						
60 mg	3 (15%)	1 (33%)	0	0	2 (66%)	1 (33%)
80 mg	17 (85%)	9 (53%)	3 (18%)	1 (6%)	4 (24%)	13 (76%)
Age						
Age ≤ 60^a^	10 (50%)	6 (60%)	1 (10%)	1 (10%)	2 (20%)	8 (80%)
Age > 60^b^	10 (50%)	4 (40%)	2 (20%)	0	4 (40%)	6 (60%)
AML diagnosis						
Newly diagnosed	12 (60%)	7 (58%)	3 (25%)	1 (8%)	1 (8%)	11 (92%)
Relapsed/refractory	8 (40%)	3 (38%)	0	0	5 (63%)	3 (38%)
European LeukemiaNet risk group						
Favorable	3 (15%)	3 (100%)	0	0	0	3 (100%)
Intermediate I/II	9 (45%)	4 (44%)	2 (22%)	0	3 (33%)	6 (67%)
Adverse	8 (40%)	3 (38%)	1 (13%)	1 (13%)	3 (38%)	5 (63%)
Total	20 (100%)	10 (50%)	3 (15%)	1 (5%)	6 (30%)	14 (70%)

^a^7 newly diagnosed; 3 relapsed/refractory

^b^5 newly diagnosed; 5 relapsed/refractory

Nineteen (95%) patients completed induction therapy. One patient (5%) died during induction. One patient who failed to respond to initial induction transferred care to another institution to enroll in another clinical trial. No patients withdrew from the study due to AEs. Of the 14 patients who responded, 5 proceeded to consolidation, 8 underwent allogeneic HCT (of which 1 was initially in consolidation prior to transplant), and 1 relapsed soon after achieving CRi and enrolled in another clinical trial. No dose reductions were needed during induction, but two patients required a reduction of selinexor from 80 mg to 60 mg during cycle 1 and cycle 2 of consolidation due to fatigue and nausea. The number of patients undergoing allogeneic HCT was 40% (*n* = 8/20) for all patients and 57% (*n* = 8/14) for responding patients.

Of note, 5 (25%) patients had *FLT3*-mutated AML (3 had ITD and 2 had TKD mutations) (Table 1). Four of the 5 (80%) achieved a CR. Only one patient had received a prior *FLT3* inhibitor on another clinical trial. All 5 patients went on to allogeneic HCT. Five (25%) patients had *NPM1* mutations, of which 3 also had a *FLT3* mutation; all 5 patients achieved CR. While these numbers are small, further study of the effect of selinexor/HiDAC/Mito on the *FLT3* mutation may prove fruitful [23].

### Survival

**Fig. 2:**
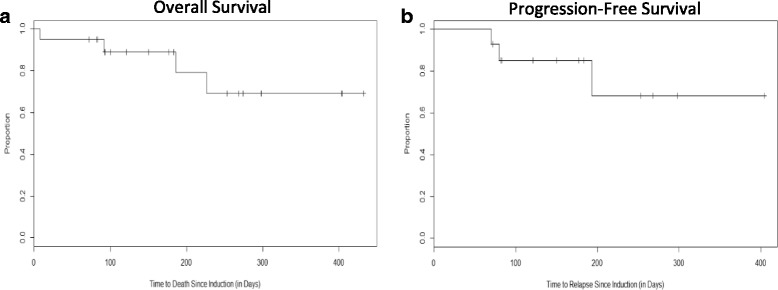
Kaplan-Meier curves depicting patient survival and relapse since the start of induction. **a** Overall patient survival (*n* = 20). **b** Relapse-free survival (*n* = 14)

At the time of this analysis, 80% (*n* = 16/20) of patients were alive and the median survival has not been reached. The median follow-up time for all patients was 6.0 months (range, 8 days to > 14 months), and one-year survival was projected at 69% (Fig. 2a). Of the 4 total deaths, 1 died during induction (on day 8), 2 patients who did not respond died from disease progression, and 1 responded but died after relapse. The projected 1-year progression-free survival was 68% (Fig. 2b).

### Correlative molecular studies

**Fig. 3:**
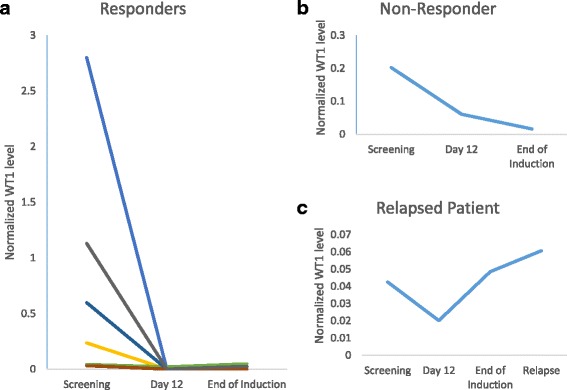
WT1 levels at set time points in induction and at relapse. WT1 levels were assessed in bone marrow samples from 8 patients (7 CR/CRi/PR, 1 TF) shown here at the start of induction, at the day 12 nadir, and the end of induction defined as either count recovery or day 56, and at relapse if applicable. WT1 levels were normalized against the control ABL level. **a** WT1 levels of 7 patients who achieved a response. Screening baseline range 0.03–2.80; day 12 range 0.00–0.02; end of induction range 0.00–0.05. **b** One patient did not respond and maintained detectable WT1 at all time points (0.20, 0.06, 0.02). **c** One of the 7 responders displayed in **a** initially achieved an incomplete response but then relapsed. This patient had detectable WT1 at all time points (0.04, 0.02, 0.05, 0.06 at relapse)

WT1 levels were assessed as a marker of MRD by the serial collection of peripheral blood and marrow aspirates at baseline, at the day 12 nadir time point during induction, at count recovery or prior to day 56, and at relapse. WT1 levels for 8 patients are summarized in Fig. 3. All patients showed a reduction in WT1 by day 12. In the 7 patients who responded to induction treatment and had samples analyzed, WT1 level was detectable at baseline (median normalized WT1 ratio = 0.200), undetectable at day 12 (median = 0.000), and either remained undetectable or increased very slightly (median = 0.015) in the marrow at count recovery. One of the 8 patients failed to respond and retained a detectable level of WT1 at day 12 (Fig. 3b). Figure 3c highlights one patient who initially responded (achieved CRi) then relapsed; this patient also retained a detectable level of WT1 at day 12, then had an increase at the count recovery marrow, and subsequently a sharp increase of WT1 at the relapse marrow.

**Fig. 4:**
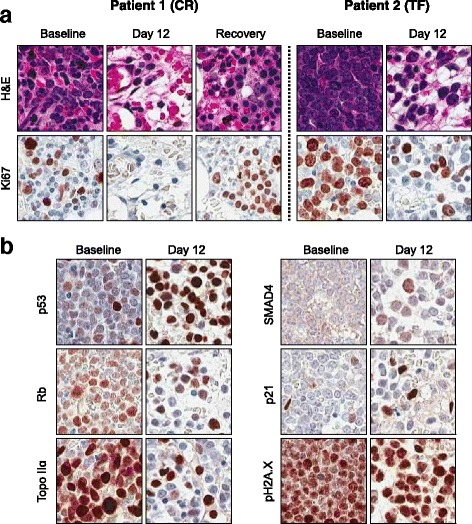
Immunohistochemistry staining of DNA damage response proteins. **a** H&E and Ki67 staining from a CR patient (left panels) and a TF patient (right panels) demonstrate characteristic bone marrow morphology and cell proliferation profile. In both CR and TF patient samples collected at baseline, tumor cells actively proliferate as shown by intensive Ki67 staining. In CR patient, complete remission of tumor cells was achieved at day 12 with few dividing cells visible; at recovery stage, high level of Ki67 staining in clustered cells is typical of active hematopoiesis. In TF patient, although complete remission was not achieved, tumor cell density and cell proliferation (Ki67) were significantly reduced in day 12 bone marrow. **b** Increased nuclear staining of major TSPs p53, SMAD4, Rb, and p21 were demonstrated in bone marrow biopsy samples collected from a TF patient at baseline and at day 12. Less cells stained positive for topoisomerase IIα after induction at day 12, suggesting reduced cell proliferation. Increased phosphorylated γH2A.X (Ser 139) staining after induction indicate more DNA damage at day 12

To demonstrate the activity of HiDAC/Mito and selinexor, IHC staining of DNA damage response proteins was performed on core biopsy slides at baseline, at the day 12 nadir, and at the count recovery marrow or day 56. Marked reductions in the proliferation marker Ki67 [24] were observed in the remaining tumor cells on the post-treatment biopsy slides of one patient who achieved CR and one who failed to respond (Fig. 4a). Staining for major TSPs p53, SMAD4, Rb, and p21 showed very little nuclear staining in the pre-treatment sample with an increase in nuclear staining intensity in the post-treatment marrow biopsy slides (Fig. 4b). This is consistent with the known effect of selinexor on tumor cells [10].

## Discussion

The combination of selinexor with HiDAC/Mito for induction chemotherapy in AML is a feasible, tolerable, and effective regimen. This study resulted in an ORR of 70% and a low induction death rate of 5%. This phase I study demonstrates that this first-in-class SINE compound has an acceptable toxicity profile at the tested doses of 60 and 80 mg. The MTD was not determined, and no DLT was observed in the dose escalation phase. However, a frequent hematologic toxicity was prolonged thrombocytopenia and delayed neutrophil recovery. Four patients received filgrastim to promote count recovery, but this did not shorten count recovery time. Moreover, studies on filgrastim have not demonstrated a consistent benefit with respect to lower rates of infections and improved survival [25, 26]. While patients eventually recovered blood counts, one patient with secondary AML had a hypocellular bone marrow prior to induction therapy and hence failed to recover blood counts by day 56. Prior studies of selinexor alone have not reported any adverse effects on hematopoietic function [7, 12, 27]. Nevertheless, based on this experience, patients with a hypocellular marrow prior to induction should receive this regimen with particular caution, and dose-reduction of selinexor should be considered based on marrow cellularity and age.

Commonly reported non-infectious toxicities included diarrhea, anorexia, electrolyte abnormalities, nausea, vomiting, and fatigue, consistent with prior selinexor studies [12, 27–29]. The Larson et al. study [16] of HiDAC/Mito alone in high-risk AML patients offered some insight into the independent contribution of selinexor to the toxicity profile. The study reported 64% of patients with neutropenic fever (vs 70% in our study), 12% with diarrhea (vs 40%), 14% with bacteremia (vs 25%), and 10% with cardiac toxicity (vs 25%), an expected toxicity in the setting of Mito exposure [30]. Additionally, more febrile neutropenia and bacteremia were observed likely due to the longer period of myelosuppression. Despite the higher number of toxicities, the majority were low grade and manageable with supportive therapies. The single case of cerebellar toxicity could be attributed to HiDAC [31, 32], as the patient was 61 years old at the time and had reduced glomerular filtration rate, yet it is worth noting that this was also observed in other phase I studies involving selinexor [7, 28]. One patient’s protracted nausea and vomiting which required TPN was assessed as selinexor-related [12, 27]. While GI toxicities were a common reason for patient withdrawal or dose modifications in other selinexor studies [12], no patients withdrew or needed modification to the dosing during induction due to toxicities. Those who proceeded to consolidation therapy continued to tolerate the regimen, although dose reductions were needed in a couple of cases, suggesting that the 80-mg dose was less tolerable for extended longer-term use. Based on these findings, the recommended phase 2 dose of selinexor is 80 mg (~ 50 mg/m^2^) twice a week for four doses in combination with HiDAC/Mito for induction therapy.

The major limitations of this study were the small sample size, heterogeneous population, and short duration of follow-up that precluded drawing any definitive conclusions. Moreover, new data on a second-generation XPO1 inhibitor reported better tolerability due to decreased CNS penetration of the drug as compared to selinexor in the pre-clinical setting [33]. The development of a second-generation agent offers additional avenues for further study as a single agent or in combination therapy.

## Conclusion

The novel class of XPO1 inhibitors have demonstrated effectiveness in disrupting cellular mechanisms that promote tumorigenesis. Selinexor is one such agent within this class that has been well studied as a single agent in AML. We demonstrated that selinexor can be feasibly combined with an existing AML regimen HiDAC/Mito, and this combination is safe with a tolerable toxicity profile. This study reported an ORR of 70% with a low induction death rate of 5%. Given the potential promise of the preliminary data, further study is needed in a large phase II trial to see if these trends would continue. The RP2D of selinexor is 80 mg (~ 50 mg/m^2^) twice a week for four doses in combination with HiDAC/Mito.
